# Incongruent dissolution of silicates and its impact on the environment: an example of a talc mine

**DOI:** 10.1038/s41598-023-50143-y

**Published:** 2023-12-18

**Authors:** Alicja Kicińska, Radosław Pomykała

**Affiliations:** 1 Faculty of Geology, Geophysics and Environmental Protection, Department of Environmental Protection, AGH University of Krakow, Mickiewicza 30 Av., 30-059 Kraków, Poland; 2 Faculty of Mining and Geoengineering, Department of Environmental Engineering, AGH University of Krakow, Mickiewicza 30 Av., 30-059 Kraków, Poland

**Keywords:** Ecology, Environmental sciences

## Abstract

The paper analyzes the process of incongruent dissolution of silicates taking place in close proximity to a talc mine. The chemical and phase composition as well as the concentrations and mobility of potentially toxic elements (PTE) in research material with varying levels of weathering were determined using instrumental (XRF, XRD) and chemical methods (extractions: BCR, *aqua regia*, water leaching, 0.05 M EDTA). It was demonstrated that the predominant minerals in the weathering crust include weathering-resistant minerals (i.e. quartz and muscovite) and secondary minerals (kaolinite, illite and interstratified minerals, vermiculite/chlorite) and that the predominant processes are hydrolysis and oxidation. The weathering process has an impact on the complexity of the chemical and mineral composition and the diverse structure of the weathering crust. A layer of Fe and Al oxides and hydroxides forms in the upper part of the weathering crust, while the amount of silica decreases. Low-mobility elements (i.e. Si, Al and Fe) react on the phase separation surface, causing the formation of clay minerals (i.e. vermiculite, montmorillonite) or Al and Fe hydroxides (e.g. goethite). The duration of weathering causes an increase in the content of PTEs in solid materials: multifold in the case of Cr (15), Ba (9), Pb (7), Zn (6) and considerably lower in the case of V (1.3), Sr (0.8) and Co (0.4). It was demonstrated that PTEs co-occur in several chemical fractions in the weathering crust and that the highest share of their total concentration are cations incorporated in the crystal lattice of minerals and bound by strong crystalline bonds (F4 46%). The lowest share was observed for the reducible fraction (9%) and the share of the oxidizable fraction was 29% The most mobile cations connected by the weakest bonds comprised only 16% of the total concentration. Based on the content of the readily soluble fraction of PTEs, it was concluded that the degree of weathering does not increase the environmental risk, but actually reduces it for Cr, Cr and Pb. The obtained Ecological Risk Index (*ERI*) values indicate that the ecological risk associated with the elements tested is low for the entire area, which means that natural weathering processes do not have any impact on environmental pollution.

## Introduction

Silicates and aluminosilicates are the most abundant group among minerals found in the Earth's crust^1^. They are composed of ionic silicon (Si^4+^), oxygen (O^2−^) and base metals (i.e.: K^+^, Na^+^, Ca^2+^, Mg^2+^ and Al^3+^). They sporadically contain hydrogen (H^+^), chlorine (Cl^−^) or the sulfate anion (SO_4_^2−^). They may occur in a hydrous form, containing H_2_O particles. The classification of silicates and aluminosilicates is based on the level of anion (SiO_4_^4−^) condensation, which reflects the ability of two or more identical silicate units (tetrahedra) to combine together to form more complex structures^2,3^. Based on the O:Si ratio, silicates and aluminosilicates are classified into the following types: ortho silicates (4:1), pyro silicates (3.5:1), cyclic silicates (3:1, 2.5:1), chain and double-chain silicates (3:1, 2.75:1), sheet silicates (2.5:1) and three dimensional silicates (2:1). Sheet anions consist of interconnected six member rings of SiO_4_^4−^ tetrahedra, with excess O^2−^ charges binding K, Na, Ca, Mg and Al cations. This results in hexagonal or pseudohexagonal crystals with excellent cleavage^4,5^. This type of minerals includes talc Mg_3_[(OH)_2_ǀSi_4_O_10_] or kaolinite Al_4_[(OH)_8_ǀSi_4_O_10_]. Talc is formed of poorly developed pseudohexagonal plates, whose shape resembles scales or slates; they can also be fibrous or compact. The theoretical chemical composition of talc is SiO_2_ (63%), MgO (32%) and H_2_O (5%). This mineral often contains admixtures of Fe^2+^, Cr^3+^ and F^-^. In turn, kaolinite is composed of SiO_2_ (46.5%), Al_2_O_3_ (39.5%) and H_2_O (14%). Compared to talc, the amount of admixtures in kaolinite is small. Nevertheless, the content of Fe^2+^ may reach 1%. This mineral is the basic raw material for the production of fine ceramics (porcelain, porcelite) and refractories. Silicates and aluminosilicates are commonly found in nature and play and important role in the environment (e.g. they are the main inorganic constituent of soils) and as such regulate many physical and chemical properties of environmental components, including soil fertility^6–11^. Due to their fine grain size (about 1 µm), many of them are classified as clay minerals. Silicates and aluminosilicates are basic raw materials for the ceramic, rubber and cosmetic industries. Thus, since early 1970s, they have been the subject of numerous studies^5,11–14^.

From the geological perspective, clay minerals are secondary weathering minerals and, quantity-wise, they comprise an important constituent of sedimentary rocks. Their genesis may vary. Stoch^15^ distinguished 3 genetic types of clay minerals: allogenic (otherwise known as clastic), transformed and newly-formed. It is believed that they are formed as a result of chemical weathering of rocks caused by hydrothermal factors^16,17^. The main weathering processes involved are: (*i*) hydrolysis and incongruent dissolution of silicate rock-forming minerals, (*ii*) transformation of the sheet silicate structure, usually combined with ion exchange with the surrounding environment, (*iii*) synthesis from ionic solutions, and (*iv*) colloidal re-crystallization. Out of these processes, the first two play the most important role in the environment^18–20^.

Dissolution of silicate minerals is a complex process^21–23^. Some of the minerals transit into the solution, while others form insoluble substances with water (e.g. Al hydroxides, kaolinite, sericite). Dissolution of a solid phase in a liquid, which involves a transit of its constituents into the solution in different proportions than the initial ones and leads to the formation of a new phase with a different chemical composition is called incongruent dissolution^24^. This is a chemical weathering process commonly occurring in nature, as it takes place at the boundary of phases (solid and liquid), i.e. whenever minerals (silicates and aluminosilicates) come into contact with water. Silicate and aluminosilicate weathering leads to the formation of hydrosilicates and hydroaluminosilicates and the precipitation of silica in the form of H_4_SiO_4_ (true solution) or SiO_2_ (colloid). In marine environments, secondary hydroaluminosilicates are formed, i.e. illite (K, H_3_O)Al_2_[(OH)_2_ǀAlSi_3_O_10_], glauconite (K, Na, Ca)_1.2–2.0_(Fe^3+^, Al, Fe^2+^, Mg)_4_[(OH)_4_ǀAl_1.0–0.4_Si_7.0–7.6_O_20_]∙nH_2_O or chamosite (Fe^3+^, Fe^2+^)_3_[(OH)_2_ǀAl_1.2–2_Si_2.8-2_O_10_]∙(Mg, Fe)_3_(OH)_6_.

In the context of the above-mentioned facts, abundant research material has been collected at the site of silicate ore extraction: unweathered material taken directly from the wall of the mined deposit, highly weathered material taken from the surroundings of the mine and slightly weathered material taken from the post-mining waste landfill, in order to trace the process of transformations and changes of the silicates and aluminosilicates contained in the material. Specific goals of the present research were to:

i) Analyze and describe the transformation of clay minerals following incongruent dissolution of sheet silicates;
ii) Determine the content of potentially toxic elements (PTE) in mineral complexes formed at individual stages of ore mineral transformation;
iii) Determine PTE mobility associated with changes in the persistence of clay minerals understood as the disintegration of the spatial network of silicates and aluminosilicates;
iv) Conduct an assessment of environmental risk associated with the presence of ion-exchange and phytoavailable PTE fractions in material with differing levels of weathering.

## Research area

**Figure 1:**
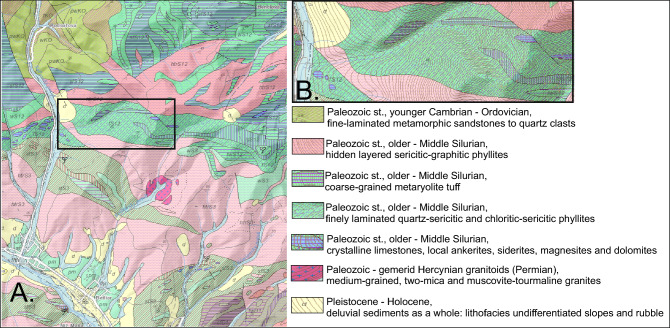
Part of geological map of Slovakia^59^ 1:50,000. (**A**) Slovak ore mountains, eastern part, (**B**) valley of Bindikovsky stream. All data available for download is subject to the Creative Commons BY 4.0 International License. Bratislava: State Geological Institute of Dionýz Štúr, 2013. Available online: http://apl.geology.sk/gm50js.

The area chosen for this research is located in Central Europe (Slovakia, EU) in the Inner Western Carpathians, more specifically, in a segment of the Variscan orogen deformed by the Alpine–Carpathian orogeny called the Gemericum^25^. Within this unit, there are highly metamorphic Paleozoic formations, mainly finely laminated quartzite sandstones (Cambrian-Ordovician), sericite-graphite and chlorite-sericite phyllites (Silurian) and coarse tuffs (Silurian) with locally occurring crystalline limestones, magnesites, dolomites and hydrothermal siderites (Silurian). Also, outcrops of muscovite-tourmaline granites can be found locally (Permian). In river valleys, one can commonly find Holocene diluvia material in the form of debris accumulated from nearby slopes (Fig. 1). Over the centuries, rock and ore mining developed in this region, exploiting the rich Zn–Pb, Cu, Ag, Au, Sb ores and other precious ores. Furthermore, hydrothermal talc deposits are regionally mined. These are found in metamorphic rocks, especially in contact zones between acidic magmatic rocks and carbonate rocks, i.e. dolomites or dolomitic limestones. The size of these deposits is substantial. The Gemerska Poloma deposit, which is found in the contact zone between granites and phyllites, is divided into three parts: West (ore: 1.6 million tons of talc), Middle (> 3.5 million tons) and East (> 10.0 million tons). The deposit was discovered in the 1980s and is one of the largest talc deposits in the world. Gemerská Poloma belongs to the Rožňava district, which is part of the Košice region. It is one of the largest villages in Rožňava^26^.

## Research materials

**Figure 2:**
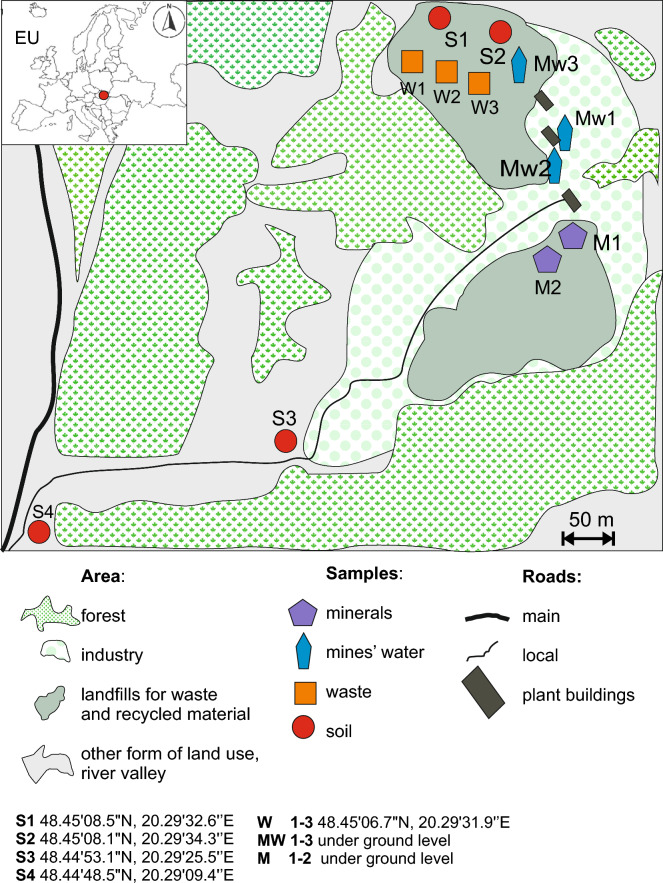
Sampling locations. Maps compliled and created by authors using the CorelDRAW2020 ver. 22.0.

The research material was collected in October 2022. Four types of samples were collected for analysis. These were (Fig. 2):

i) 2 samples of unweathered rock material (symbols: M1, M2) collected directly from a recently mined underground adit wall, comprising 5 single portions, weighing about 1 kg each.
ii) 3 samples of mine water, 2 of which were collected below the ground surface (symbols: MW1, MW2). Those were water seeps from fissures located in underground mine passages. One sample (MW3) was collected from a collection pond located on the ground surface in close vicinity (100 m) to the adit entrance. The pond is used for storing mining water that is collected and drained to the land surface. Each of the samples was collected into two separate 250 ml PCV containers (6 bottles in total), with one of the doublets fixed with concentrated HNO_3_. For the time of transportation to a laboratory, the samples were placed in a fridge at about 5 °C.
iii) 3 samples of fragmented, slightly weathered mining material collected from spoil tips (symbols: W1, W2, W3) with a varying grain size (diameter in mm: > 2, 2–0.5, < 0.5, respectively). The overall sample comprised 5 portions collected from different parts of the spoil tip. Each of the samples weighed about 0.5 kg. The material was secured and placed in PCV bags.
iv) 4 samples of highly weathered material (symbols: S1–S4) collected from the near-surface layer of soil profiles (collection depth: 0–20 cm BGL). Each sample comprised 5 portions collected from the corners and the central point of a 0.5 × 0.5 m square, weighing 0.2 kg each. Two of them, samples S1 and S2, were collected in close proximity (about 50 m) to spoil tips built of tailings and to the plant processing the extracted mineral (about 75 m). The other two samples (S3 and S4) were collected from areas located at a greater distance from the processing plant (300 m and 600 m, respectively). It needs to be mentioned that topography-wise, samples S3 and S4 were collected from a substantially lower level than the other two.

Overall, the research material comprised 51 field samples, of which 45 were bulk solids and 6 were aqueous solutions. Based on this material, 12 samples were prepared in a laboratory. These were subsequently subjected to instrumental and chemical analyses. During the field research, pH of water was measured in situ.

## Research methods

The research material collected was dried, homogenized and prepared for chemical analyses, which were carried out in the Trace Analysis Laboratory at the Department of Environment Protection, AGH—University of Krakow (Poland). During the first stage of the laboratory work, all the samples were air-dried and separated into fractions < 2 mm and > 2 mm. Next, the following parameters were determined for the latter:

i) pH of the aqueous solutions of the solid material (1:5 solid phase/solution ratio, in accordance with PN-ISO 10390);
ii) percentage of the most mobile cations of PTEs leached with an aqueous solution (1:10 solid phase/solution ratio);
iii) phytoavailable fraction of selected elements using a single-step 0.05 M EDTA extraction (1:10 solid phase/solution ratio);
iv) content of PTE cations associated with the^27,28^:
  *Exchangeable fraction* (F1)—easily soluble in an acidic environment and found in exchange positions and bound to carbonates; 0.1 M acetic acid extraction (CH_3_COOH);
*Reducible fraction* (F2)—prone to reduction. Metals bound to amorphous Fe and Mn oxides/oxide-hydroxides; 0.1 M hydroxylammonium chloride extraction (NH2OH∙HCl);
*Oxidizable fraction* (F3)—prone to oxidation. Metals bound to organic matter or sulfides; hydrogen peroxide extraction (H_2_O_2_) and 1 M ammonium acetate extraction (CH_3_COONH_4_);
  *Residual fraction* (F4)—element forms bound mainly in silicates; *aqua regia* extraction (HCl:HNO_3_).
v) pseudo-total PTE content using a mix of concentrated acids (38% HCl + 65% HNO_3_ in a 3:1 ratio), in a mineralizer at 130 °C.
  The chemical and phase composition of the bulk material was determined using instrumental methods (XRF and XRD, respectively).
vi) The phase composition was determined with X-ray diffractometry (XRD), using the Debye-Sherrer method of powder diffraction. X-ray images of all the samples were registered with a Rigaku MiniFlex 600 X-ray diffractometer using the following parameters: Cu_Kα_ radiation, graphite monochromator, tube voltage 40 kV, tube current 20 mA, 2Θ angle range 2–72°; 2Θ step 0.05°, number of counts 1 s/step. The values of the interplanar spacing obtained from the X-ray images were used to identify mineral phases in the samples analyzed based on data included in the ICDD (International Centre for Diffraction Data) catalogue and XRAYAN software. To identify clay minerals, samples saturated with ethylene glycol were prepared and then roasted at 560 °C.
vii) X-ray fluorescence (WD-XRF ZSX Primus II Rigaku spectrometer, Rh tube) was used to determine the chemical composition of the powdered material by identifying spectral lines and any potential line coincidences. The analysis was conducted in the fluorine-uranium range (F-U) using SQX Calculation software (fundamental parameter method). The content of the elements identified was normalized to 100%.

PTE concentrations in the solutions were determined in an accredited hydrogeochemical laboratory (certificate of accreditation PCA no. AB1050) at AGH University of in Kraków, using the inductively coupled plasma—mass spectrometry (ICP-MS) method. Precision of quantification for Ba, Co, Cr, Cu, Pb, Sr, V and Zn was 10% and accuracy ranged from 95 to 105% (Table 1) DL of the instrument was 1∙10^−3÷5^ mg/dm^3^. The control system of the analyses (QA/QC) was in line with the standard PN-EN ISO 17025^29^ using blank samples, doubled samples (min. 25%) and marked samples in each series of determinations. Furthermore, the Standard Reference Material (CRM048, lot: LRAB1604) was analyzed. Here, the differences of the Al and Mg determinations did not exceed 15%, and those of the rest of the elements 7%. The results were statistically compiled using Excel 2013 and Statistica *ver*.13.3.

**Table 1:** Pseudo-total concentration of PTE in samples with an increasing degree of weathering.

Degree of weathering	Sample	Ba	Co	Cr	Cu	Pb	Sr	V	Zn
		mg/kg							
Zero	M1	6.06	15.13	1.62	233.74	5.41	10.43	11.77	12.93
	M2	9.33	10.42	0.81	8.00	3.84	9.87	12.14	9.04
Medium	W1	6.11	27.64	2.00	107.63	6.14	11.46	13.53	6.39
	W2	7.17	4.76	1.23	5.27	5.10	12.91	12.49	10.07
	W3	36.55	5.90	7.09	32.52	9.50	49.87	12.07	29.36
High	S1	103.57	32.33	17.56	47.86	39.14	10.75	29.62	130.94
	S2	104.10	16.82	25.63	43.91	73.04	12.21	36.54	87.59
	S3	64.45	9.21	21.13	17.49	15.22	39.48	29.46	40.67
	S4	60.85	14.38	14.64	181.15	32.44	9.70	17.03	70.50
Reference material (RM)		249	57	186	77	141	378	70	768
CRM048/LRAB1604 (Recovery *%*)		253 (98)	60 (95)	179 (104)	79 (97)	145 (97)	359 (105)	72 (98)	770(100)
Clay rocks^1^		500–800	11–20	60–120	40–60	20–40	30–450	80–130	80–120
Fine-grained sedimentary rocks (pelithic)^2^		207	12	36	25	23	98	38	43
Sedimentary medium-grained rocks (sandstone)^2^		127	6	26	7	10	109	22	22
Acidic igneous rocks^1^		400–1200	1–15	4–25	5–30	10–25	60–400	40–90	40–100
Soils (various type)^1^		20– 520	5.5–12	12–740	5–23	13–26	10–200	15–115	35–100

^1^Kabata-Pendias & Pendias^53^.

^2^Kicińska^57^.

## Results and discussion

### Weathering and transformation of clay minerals

**Figure 3:**
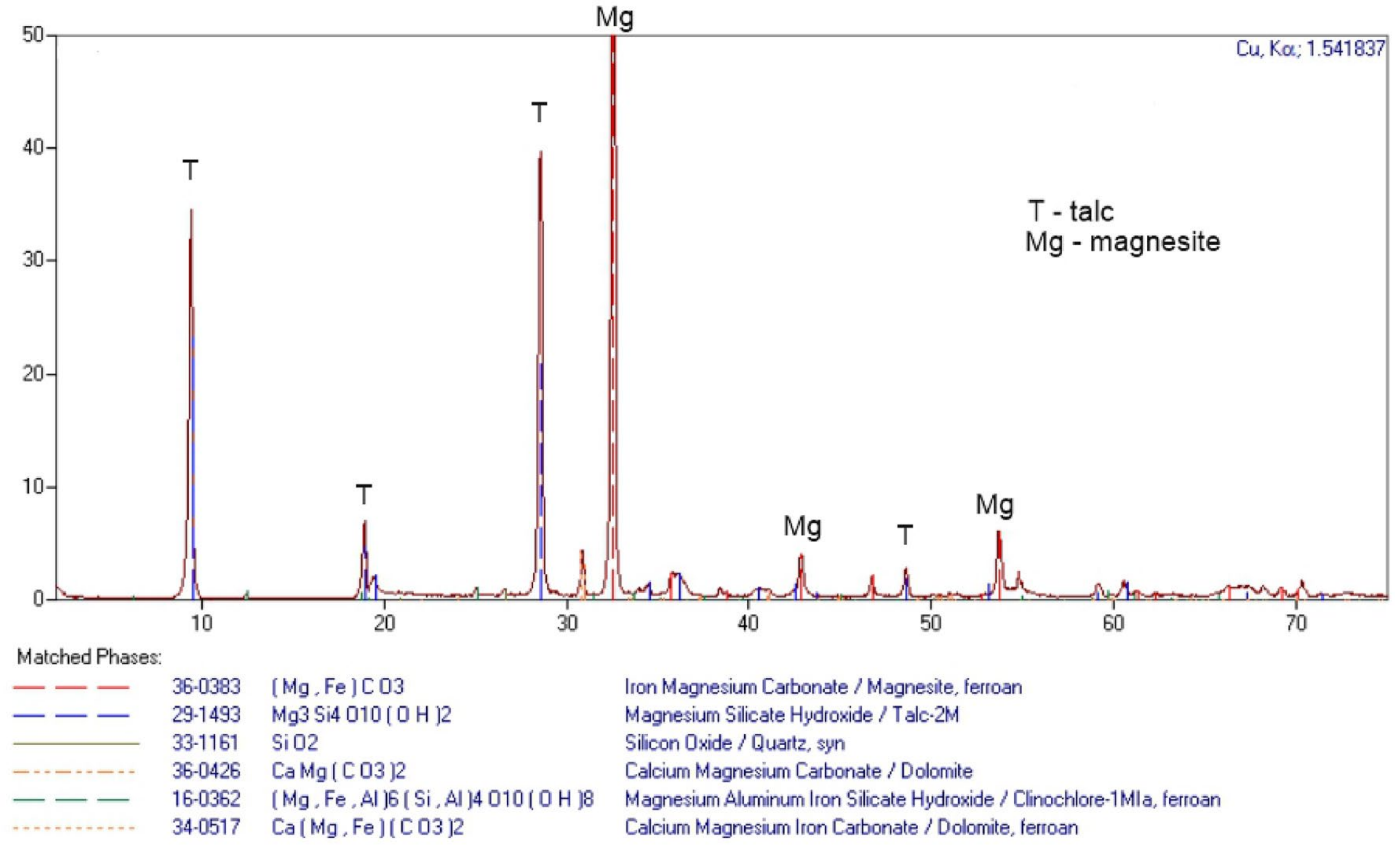
XRD patterns of extracted materials (sample M1, Fig. 2).

The registered spectrum (XRD) of the unweathered material (Fig. 3) collected directly from the mined walls showed intense and sharp peaks from the reflections of the two predominant minerals, i.e. talc (Mg_3_[(OH)_2_ǀSi_4_O_10_]) and magnesite (MgCO_3_). There were also traces of quartz, dolomite and clinochlore, but these were not significant amounts, comprising up to 5%.

From the chemical point of view, this talc and magnesite mix consists of the compounds of (in wt.%): Si (50.7), Mg (40.9), Fe (4.4), Ca (2.3), which constitute 98.8%. The compounds of the following elements are found in considerably smaller quantities (in wt.%): S and Al (0.3 each), F, Mn and Mg (0.2 each), P and K (0.1 each). The compounds of the following elements are found in trace amounts (i.e. < 0.1 wt.%): Na, Cl, Ni, Cu, Sr, Y and Zr (not more than 0.5% in total).

**Figure 4:**
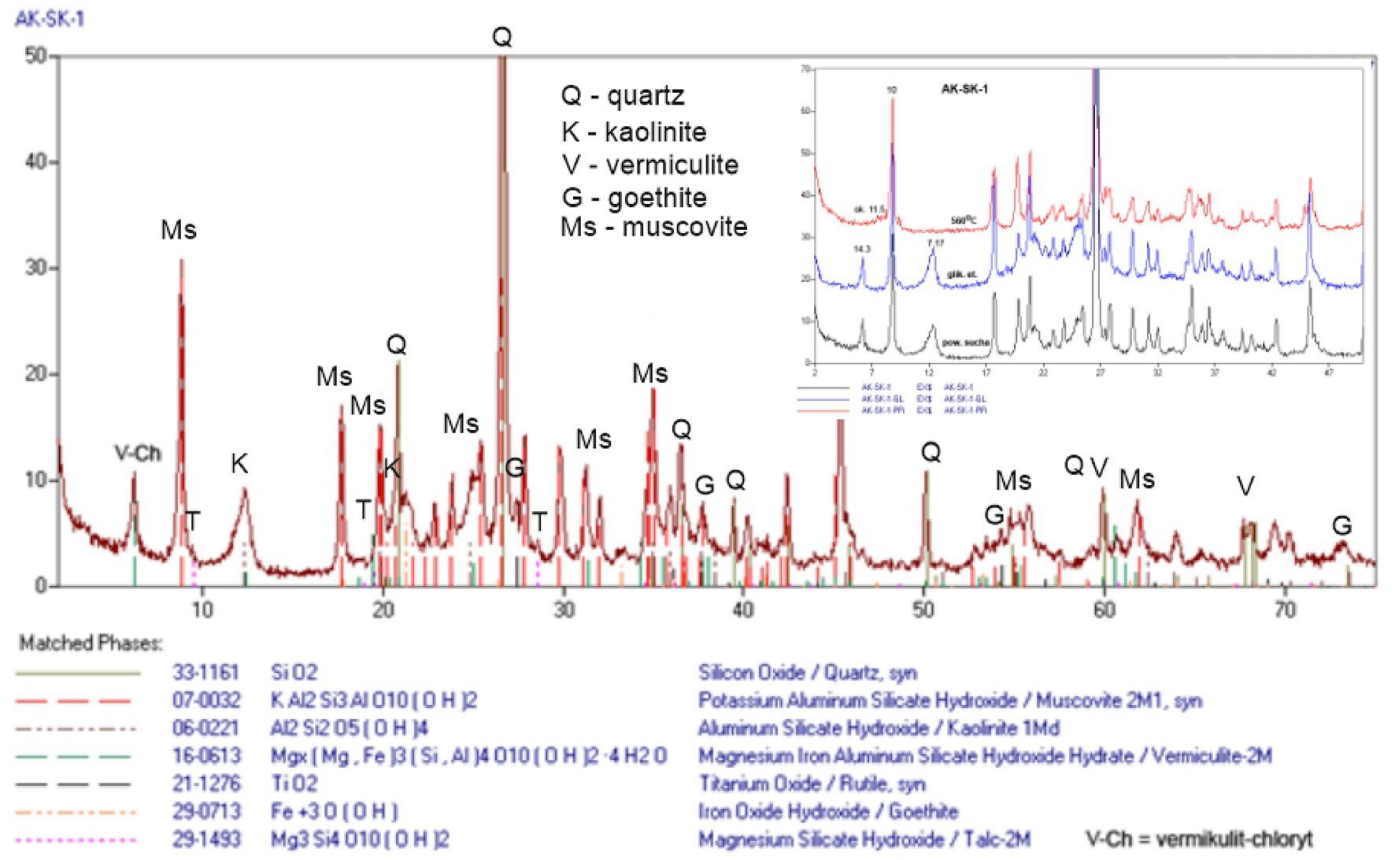
XRD patterns of weathered sample (S1, Fig. 2).

The phase analysis of the material subjected to long-term physical and chemical weathering processes (Fig. 4) revealed that the predominant mineral phase included quartz (SiO_2_) and muscovite (KAl_2_[(OH,F)_2_ǀAlSi_3_O_10_]), as these are weathering-resistant minerals forming eluvial deposits. Other detected minerals included kaolinite Al_4_[(OH)_8_ǀSi_4_O_10_] and an interstratified mineral—vermiculite/chlorite (Mg,Fe^3+^,Al)_3_[(OH)_2_ǀAl_1.3_Si_2.7_O_10_]∙Mg_0.3_(H_2_O)_4_, which indicates a weathering zone with intense hydrolysis and oxidizing conditions^4,15^. There were also trace amounts of goethite (α-FeOOH), which may result from the heavy infiltration of the weathered soil by rainwater. This, in turn, contributes to intensive migration of both alkali and poorly soluble silica^5^. The detected presence of rutile (TiO_2_) in the weathering crust may stem from the high resistance of this mineral to water. Usually, simple minerals with low migration intensity (as is the case with Ti) are more resistant to hydrolysis. Furthermore, the material also contained a trace amount of talc.

From the chemical point of view, the weathered material consists of the compounds of (data in wt.%): Si (49.2), Al (31.5), Fe (10.0), K (5.4), Mg (1.2) and Ti (1.1), constituting 98.4 wt.%. The compounds of the following elements were found in considerably smaller quantities (in wt.%): Na (0.5), P, Ca and Mn (0.2 each) and Ba (0.1). The compounds of the following elements were found in trace amounts (< 0.1 wt.%): S, Cl, V, Cr, Co, Ni, Cu, Zn, Ga, Rb, Sr, Y, Pb and Zr (about 0.4% in total).

Based on the analyses conducted, it was found that the weathered material:

- has a decidedly broader elemental spectrum as compared to the primary material. This is due to the polygenetic, mixed material from close and more distant areas, originating from both natural weathering processes and deposition caused by aeolian and water transport^30,31^. What is also important is the role of biological weathering (associated with the presence of fungi, bacteria, algae and other organisms, like vertebrates). Microorganisms make such elements as Fe, Ca and Si transit as anions into the solution and form complexes with organic secretions^15,32^. The role of the flora in weathering processes, associated with the formation of such complexes has been described by numerous authors^33–37^.
- contains a more numerous group of major elements, i.e.: Al, K, Ti, Si and Mg, whose presence is associated with greater variability of chemical, mineral and structural composition of the weathering crust. Important factors pertaining to the predominant elements are the processes of Fe migration of precipitation as well as redox and pH changes^38^. As mentioned before, the predominant processes in the upper part of the weathering profile are hydrolysis and oxidation. These processes have such high intensity that, as a result, silica is removed and a layer of Fe and Al oxides and hydroxides is formed. In turn, the content of K (5.4 wt.%) is associated with the presence of muscovite and the weathering of acidic magmatic rocks, namely granites found in the ground surrounding the mine. Rutile (TiO_2_) is an accessory mineral in magmatic rocks. Its main constituent—titanium—does not migrate due to poor solubility. Its chemical activity also depends on the environmental pH and redox conditions^39,40^.
- contains more minor elements, i.e. Na, P and Ba (0.5–0.1 wt.%) and smaller amounts of Ca. The reduction in the content of Ca (from 2.3 to 0.2%) may be caused by the dissolution of susceptible minerals (mainly mafic minerals) by groundwater and their participation in the subsequent stage of clay mineral transformation or in the formation of secondary carbonate minerals. In turn, the presence of Na, P and Ba in the weathered material stems from the fact that these elements are readily mobilized by weathering processes and easily released from the weathering crust^41,42^.
- contains a more numerous group of trace elements: S, Cl, V, Cr, Co, Ni, Cu, Zn, Ga, Rb, Sr, Y, Pb and Zr (< 0.1 wt.%), which constitute admixtures in primary minerals in both acidic magmatic rocks and clay minerals (e.g. Cr, F, Ni, Co, Zn, Cl, Rb). In the case of this group of elements, the form of a given element and the already mentioned pH and redox conditions play a particular role in the mobility process in hypergenic environments^43^. As for Pb, an important role can be played by microorganisms, especially in the near-surface oxidation processes, and Pb^2+^ can be readily bound by mineral and organic components of eluvial deposits^44–46^.

In summary, following the hydrolysis of silicates and aluminosilicates, the constituents of the primary mineral structures transit into the solution in different proportions (incongruent dissolution). As described by Stoch^15^, low-mobility elements (Si, Al and Fe) react on the phase separation surface, causing the formation of clay minerals (vermiculite, montmorillonite) or Al and Fe hydroxides (goethite). The content of elements found in small quantities in primary minerals increases in the solutions (e.g. Na and K). Nevertheless, solid products, insoluble under certain conditions (at a given temperature, pressure, pH and redox conditions) are also formed during silicate and aluminosilicate dissolution.

Undoubtedly, the dissolution of silicates and aluminosilicates is affected by external and internal factors that include:

- Chemical composition of water, especially its pH. In the water samples collected, pH ranged from 9.46 to 11.10 (which points to a highly alkaline reaction). An additional factor that determines pH and decidedly increases solubility may be the amount of dissolved gases (including CO_2_) and organic acids,
- Chemical and mineral composition of primary minerals,
- Climatic conditions, including precipitation (water infiltration and intensity of hydrolytic product migration), and land morphology^47,48^.

In turn, the type of secondary minerals formed by weathering is affected by the chemical constituents of primary rocks, which display greater stability in a given environment, as described by Cabral Pinto et al.^49^ and Cox et al.^50^. Unstable elements are easily soluble and leached and can change the local weathering conditions, mainly understood as solution chemistry, which in turn entails the generation of chemical and mineral zonation in the weathering crust, as mentioned by Chen et al.^51^, Feng et al.^52^ and Perri^17^. Thus, it can be confirmed that soil profiles which undergo continuous soil-forming processes (i.e. physical, chemical and biological weathering) constitute the modern weathering crust^1^.

### Content of PTEs in mineral complexes formed at individual stages of ore mineral transformation

To determine differences in the content of PTEs, the research material was analyzed in the following, ascending order with regard to the impact of weathering processes.

1. Primary unweathered material (samples: M1, M2, Fig. 2)—the weathering time was assumed to be zero.
2. Slightly weathered material (samples: W1, W2, W3, Fig. 2) weathering time: about 3–6 months.
3. Highly weathered material (samples: S1, S2, S3, S4, Fig. 2) long weathering time lasting tens and hundreds of years.

PTEs selected for analysis were: Ba, Co, Cr, Cu, Pb, Sr, V and Zn. Their importance and detailed role in the geo- and biosphere have been described by Kabata-Pendias and Pendias^53^. The present paper does not discuss arsenic due to its specific role, which will be presented in our next publication.

The content of PTEs in the samples analyzed is presented in Table 1. Having calculated the mean PTE content (for all the samples collected, *n* = *45*), we obtained the following descending order (data in mg/kg): Cu (75) > Ba = Zn (44) > Pb (21) > V (19) > Sr (18) > Co (15) and > Cr (10). Comparing these values to the mean content determined for clay, acidic magmatic and sedimentary rocks (with varying degrees of grain size), it can be concluded that:

- The chemical composition of the unweathered material taken directly from the mined wall was stable and basically comparable in all the samples. On the other hand, the content of Ba, Cr, Pb, Sr, V and Zn was substantially lower than the quantities observed by other authors in clay rocks^52,54^. The content of Cu ranged from 8 to 233 mg/kg, which is the effect of the common occurrence of this element in early-magmatic deposits or granodiorite intrusions in the form of local Cu mineralization.
- The material subjected to short-term weathering showed a significantly broader content range for individual PTEs. Compared with the literature data^43,54,55^, the content of Ba, Cr, Sr, V and Zn was lower; the content of Co and Cu was higher in the coarse material; and the content of Co, Cr, Cu and Pr was comparable in the fine-grain material. The highest content of Ba (37 mg/kg), Pb (10 mg/kg), Sr (50 mg/kg) and Zn (29 mg/kg) was found in the sample with the finest grain size (< 0.5 mm). The lowest content of PTEs was found in the medium-grained material (grain size 2–0.5 mm), whose predominant constituent was silica (SiO_2_).
- In the highly weathered material, the PTE content range was the widest. The content of all PTEs analyzed in our study was comparable to the values reported by other authors, except for Ba, whose content was lower. An increased content of Co, Pb and Zn was found in sample S1, collected closest to the processing plant, whereas an increased content of Cu was observed in sample S4 taken furthest away from the plant. The highest concentrations of Ba, Co, Cr, Pb, V and Zn were found in samples taken closest to the processing plant.

**Figure 5:**
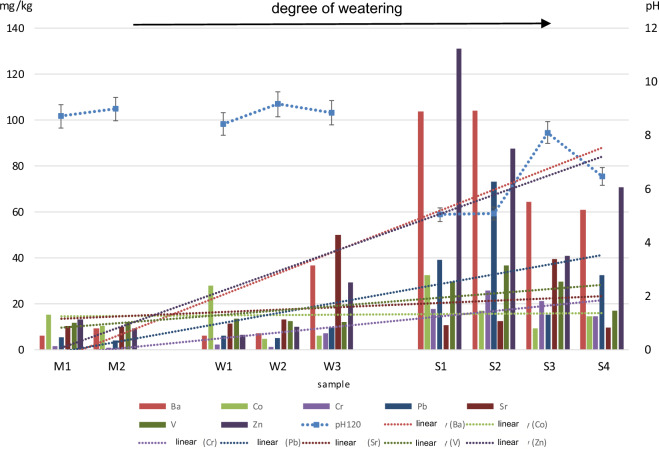
PTE concentration in the samples with an increasing degree of weathering.

Based on the results obtained, we also found that the weathering time in the region studied is associated with an increase in the content of Cr (15-fold), Ba (ninefold), Pb (sevenfold), Zn (sixfold), V (by 130%), Sr (by 80%) and Co (by 40%), and a decrease in the content of Cu by 40%. The same observation applies to major elements, namely Al, Si, Fe and K (Fig. 5).

Cations that transit into the solution during the hydrolysis of silicates and aluminosilicates are mainly those of weak (Na^+^, K^+^, Ca^2+^, Mg^2+^) and intermediate (Al^3+^, Fe^3+^, Si^4+^) ionic potential. As a result, suspensions of fragmented material derived from silicate minerals produce an alkaline reaction (Fig. 5). The pH of the aqueous solutions of the unweathered material (samples M1 and M2) was very similar and stable over time. It ranged from 9.2 ± 0.2 immediately after suspension preparation to 8.9 ± 0.2 after 2 h. These values clearly indicate that the solutions were alkaline. The aqueous solutions prepared from the slightly weathered material (samples W1–W3) were also alkaline, with their pH similar to that observed in the unweathered material (mean pH: 9.2 immediately after preparation and 8.8 after 2 h). In turn, a significant pH change was found in the highly weathered samples (symbols S1–S4). The pH values were substantially lower. The mean values calculated for all the samples were 6.3 and 6.2, respectively, which points to a clearly acidic reaction. This difference has an enormous impact on the mobility of PTE cations in the soil environment^21,36^.

### PTE mobility associated with the weathering of clay minerals

Solid phase dissolution (including silicates) depends on numerous factors^22,24,47^. The most important ones include the mineral and chemical composition and the physicochemical properties of the environment in which the dissolution takes place. The key environmental parameters are pH and redox conditions, which in turn determine the forms (also called chemical fractions) of cations and their chemical activity^40^.

**Figure 6:**
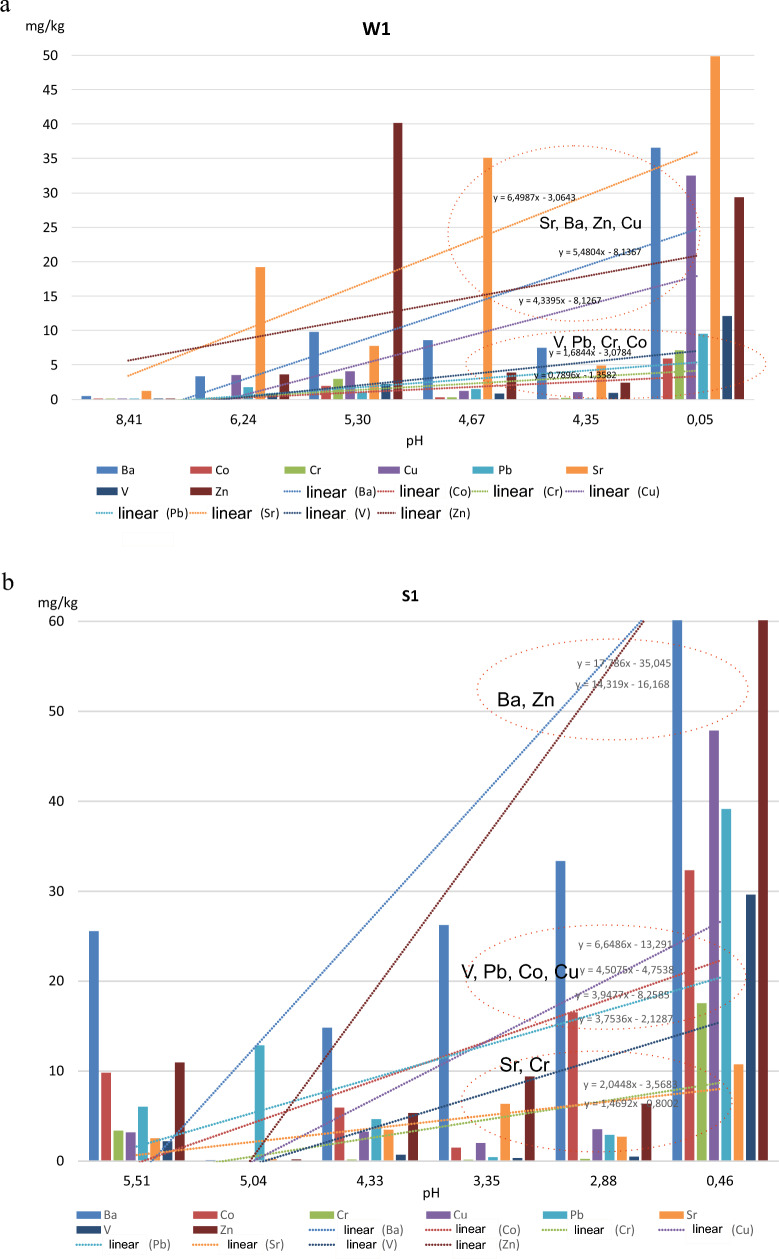
(**a**) Dissolution of PET in solutions with different pH in the material of the weathered medium (samples W1). (**b**) Dissolution of PET in solutions with different pH in highly weathered material (samples S1).

To assess the effect of pH on silicate solubility, we analyzed how the process of pH reduction (from the values observed for aqueous solutions down to the extremely low values ~ 0) affects the concentrations of PTE cations in the solutions (Fig. 6a,b).

In the case of the slightly weathered material (sample W1), the following was found: as the pH decreased from decidedly alkaline (8.41) to extremely acidic (0.05), the amount of PTE cations in the solutions increased. However, this increase was not uniform. The cations of Sr, Ba, Zn and Cu dissolved to a far greater extent in a strongly acidic environment than those of V, Pb, Cr and Co. For ∆ pH = 8.36, there was a multifold increase in the amount of dissolved cations (from the highest to the lowest values) in the following descending order (multiplication factor): Pb (~ 10,000) > Co (~ 3000) > Cr and Cu (~ 900) > Zn (~ 700) > V (~ 200) > Ba (~ 80) > Sr (~ 40).

As for the highly weathered material (Fig. 6b), a similar relationship was found for ∆ pH: 8–4.5, but the order of the elements was slightly different (multiplication factor for all samples): V (~ 30,000) > Co (~ 15,000) > Pb (~ 11,000) > Cr i Cu (~ 5000) > Ba and Zn (~ 500) > Sr (~ 50).

When comparing the amounts dissolved in the slightly and highly weathered material, we observed a clear increase in the mobility of cations over time for nearly all the elements analyzed. This was a fourfold increase for Ba, Cr, Pb and Zn, a twofold increase for V and a nearly 20% increase for Co and Cu. This increase stems from the fact that the process of weathering takes place in an open system, which is fed by new portions of rainwater (less mineralized). The very dissolution occurs at a varying pace, depends on the constant of solubility (*Ks*) and takes place at the boundary of phases. Thus, the degree of material dispersion plays a similarly important role as the redox conditions. This is why dissolution is most intensive in the near-surface layer. An inverse correlation, i.e. higher (by about 40%) amount dissolved in the slightly vs. highly weathered material was observed only in the case of Sr.

In the weathering processes, especially in acidic environments, Cu and Zn are easily dissolved and the released ions form mineral or organic-mineral complexes with anions or organic matter. These complexes are highly mobile and migrate with the solutions^31^. In the presence of sulfide ions, Zn may undergo rapid precipitation^56^. In turn, the presence of clay minerals in the weathering crust causes the sorption of Ba, Sr, Cr and Co ions. Ba easily migrates with circulating water and is leached into the soil profile. It is readily mobilized by weathering processes and undergoes rapid precipitation in the form of sulfates and carbonates. Apart from clay minerals, it is bound by sulfur compounds. As for Pb, it is slowly mobilized from sulfide compounds, usually through oxidation processes involving microorganisms. V is also easily oxidized to stable forms and does not migrate very far. Its content in all the samples of the highly weathered material was comparable and ranged from 17 to 36 mg/kg. Co is readily oxidized from Co^2+^ to Co^3+^ in weathering environments. It is mobile in oxidative acidic environments, but is not subject to extensive aqueous migration as it is bound by Fe and Mn hydroxides and clay minerals. In contrast, most Cr minerals are resistant to weathering and accumulate in the residual fraction, although the toxic form of this element (Cr^6+^) is formed in oxidative conditions and can be mobile. Clay minerals and Fe and Al hydroxides play an important role in the sorption of ions of numerous elements, including Cr and Sr^40^. In weathering processes, Sr behaves similarly to Ca and transits into the solution in the form of bicarbonate. It is easily sorbed not only by clay minerals, but also organisms with calcium skeletons^50^.

**Figure 7:**
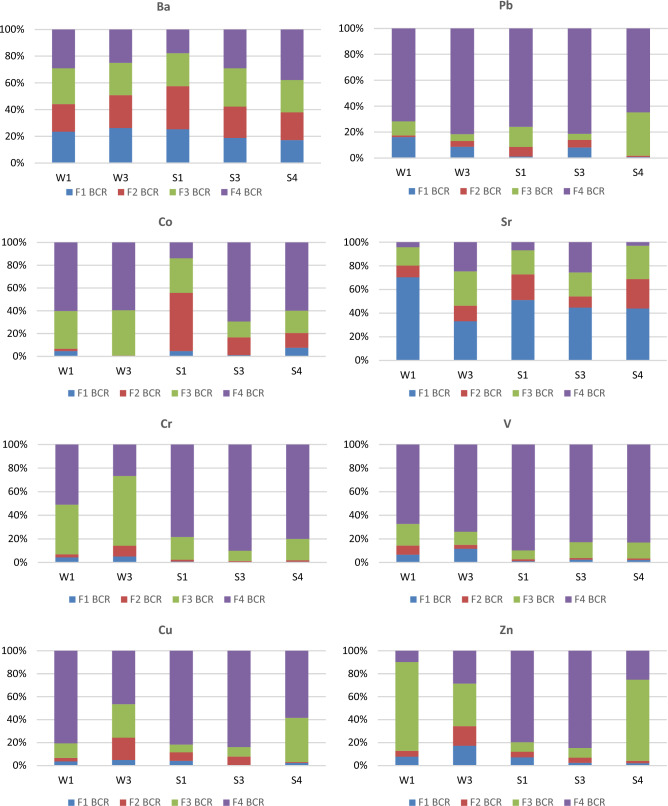
Forms of PTE bonding in medium (samples W1 and W3) and high weathered (samples S1–S4).

Another analysis in our study concerned the identification of PTE bonding forms co-occurring in the weathered material based on 4 fractions: *(F1)* ion-exchange and carbonate-bound fraction, (*F2*) reducible fraction, (*F3*) oxidizable fraction and *(F4)* residual fraction. The analysis yielded the following findings (Fig. 7):

1. In the slightly weathered material:
  The crystal lattice (F4) incorporates the highest amount of Pb (77%), V (71%), Cu (64%) and Co (60%) cations. For the other elements, the values range from 14% (Sr), 19% (Zn), 26% (Ba) to 39% (Cr);
  The oxidizable fraction (F3) constitutes from 8% (Pb), 15% (V), 21% (Cu), 22% (Sr), 26% (Ba), 36% (Co), 50% (Cr) to 57% (Zn);
  The quantity of cations in reducible forms (F2) ranges from 1% (Co), 3% (Pb), 5% (V), 6% (Cr), 11% (Cu, Sr, Zn) to 23% (Ba);
  The ion-exchange and carbonate-bound fraction (F1) constitutes from 3% (Co), 4% (Cu), 5% (Cr), 9% (V), 12% (Pb), 13% (Zn), 25% (Ba) to 53% (Sr).
  In the case of all the elements analyzed, the greatest share was observed for fraction F4. As much as 46% of PTE cations are incorporated in the crystal lattice of minerals and bound by strong crystalline bonds. The lowest share was observed for fraction F2 (reducible)—merely 9%. The share of fraction F3 was 29%, and that of the most mobile and easiest to leach (F1) only 16% of the total concentration.
2. In the highly weathered material, F4 was also the largest fraction for all the elements analyzed, as much as 58% of the total concentration, whereas the lowest share was observed for fraction F1 (exchangeable)—only 10%. The share of fraction F2 was 11%, and that of F3 20% of the total concentration.

Overall, for the entire material collected, it was found that the percentage of cations:

- Incorporated in the crystal lattice is the largest and this pertains to all the elements (except for Sr): V 85%, Cr 83%, Cu 75%, Pb 74%, Zn 63%, Co 48% and 28% Ba of the total content.
- Bound to the oxidizable fraction constitutes from 12% (V), 15% (Cr), 18% (Pb, Cu), 23% (Sr), 26% (Ba), 29% (Zn) to 48% (Co);
- Occurring in reducible forms ranges from 1% (Cr, V), 4% (Zn), 5% (Cu, Pb), 19% (Sr), 25% (Ba) to 27% (Co);
- Found in the ion-exchange and carbonate-bound fraction constitutes from 1% (Cr), 2% (Cu, V), 3% (Pb), 4% (Co, Zn), 20% (Ba) to 47% (Sr).

These findings correspond to the results obtained by other researchers for unpolluted soils^49,53,57^. Our results point to the development of natural, long-term processes in which the forms of bonding and mobility of elements depend on the weathering resistance of the minerals composed of these elements and on the water solubility of the complexes they form. In the case of the highly weathered material, it is important to remember that it contains humic substances and living organisms actively involved in weathering processes. Humification of the organic matter leads to the formation of fulvic and humic acids (large-particle polymerized compounds). Humic acids form water-soluble chelate complexes with metal cations, which in turn affects the mobility of elements. Organic matter in the form of colloids can impart hydrophobic properties to inorganic surfaces and counteract their coagulation, which in turn results in the free migration of colloidal Fe, Al or Ti hydroxides. Fulvic acids form smaller particles that readily dissolve in water and are more strongly dissociated in the solution than humic acids. Similarly to humic acids, fulvic acids form complexes with metals. These complexes play an important role in the biological life of soils, forming a storage of mineral substances and microelements. They are also believed to participate in protein synthesis and even in the biological activity of organic pesticides^23,31^.

### Assessment of the environmental risk associated with the easily soluble and bioavailable fraction of PTEs

The environmental risk assessment associated with the presence of PTEs in the material analyzed started with the comparison of the obtained values with those set out in the binding legal regulations^58^. Given the type of land development in the areas sampled, namely industrial, mining and transportation areas, the permissible concentrations of risk-causing substances (values in mg/kg): Ba—1500, Co—200, Cr—1000, Cu—600, Pb—600 and Zn—2000, have not been exceeded in any of the samples for any of the elements. In light of the strictest criterion set out for agricultural areas and allotments, namely 200, 20, 150, 100, 100 and 300, respectively, it was found that the limits for Co were exceeded in samples W3 and S1 and those for Cu in samples W3 and S4.

When comparing the pseudo-total concentrations obtained in our study to the so called natural concentrations^53,59,60^, it was found that for Co, Cr, Cu, Pb, Sr, V and Zn they were higher in all the samples collected. With Co, Cu and Sr, they were higher irrespective of the degree of weathering and in the case of Cr, Pb and V, they were higher in all samples of the highly weathered material.

The next stage of the analysis involved determination of the Risk Assessment Code (RAC), which is the quotient of the ion-exchange fraction to the total content (Table 2). Based on the easily soluble and readily mobile PTE content, it was found that:

- In the case of the slightly weathered material: the environmental risk was low for Co, Cr, Cu and V, medium for Ba, Pb and Zn and very high for Sr;
- In the case of the highly weathered material: there was no risk for Cr, the risk was low for Co, Cu, Pb, V and Zn, medium for Ba and high for Sr.

**Table 2:**
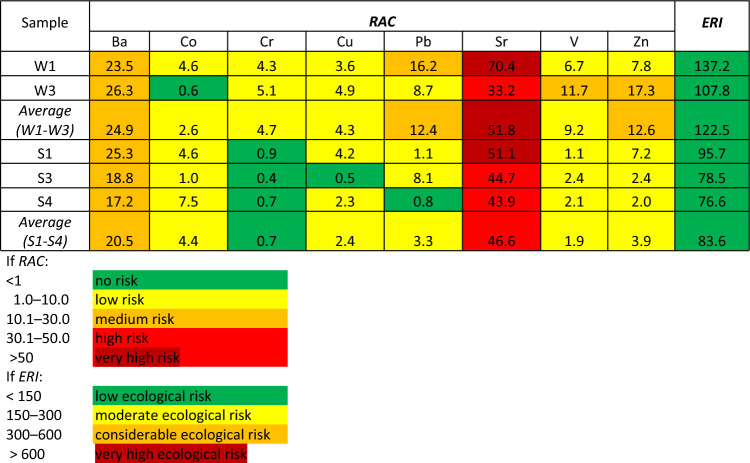
RAC and ERI calculated for material with different degree of weathering S.

The Ecological Risk Index (*ERI*) calculated as the sum of RAC values for each sample analyzed was below 150, which indicates that the ecological risk associated with the elements tested is low for the entire area.

The 0.05 M EDTA extraction allowed us to determine the quantity of PTE available to plants and organisms living in the substrate. The analysis showed that the phytoavailable content of Ba, Co, Cr, Cu, Pb, Sr, V and Zn was as follows (data in mg/kg):

- In the slightly weathered material: 0.69–3.34, 0.13–0.18, 0.16–0.24, 3.53–7.64, 0.55–1.73, 1.06–19.20, 0.48–0.83 and 1.18–3.58,
- In the highly weathered material: 2.50–18.82, 0.70–5.94, 0.10–0.17, 1.98–69.80, 1.94–4.65, 2.12–6.92, 0.48–0.70 and 2.08–5.36.

When comparing the mean content of the elements, it was found that for Ba, Co, Cu, Pb and Zn, it was higher in the highly weathered samples than in the slightly weathered material. Only in the case of Cu did the content exceed the amounts described as natural concentrations.

## Conclusions

Based on the material collected and the analyses performed, we reached the following conclusions:

1. Given the intensity of hydrolysis and oxidation processes, minerals that are predominant in the weathering crust include weathering-resistant minerals (i.e. quartz and muscovite) and secondary minerals such as kaolinite and interstratified minerals (i.e. illite, vermiculite/chlorite).
2. Over time, a much broader elemental spectrum becomes present in the weathered material, which translates into a more complex chemical and mineral composition and more diverse structure.
3. A layer of Fe and Al oxides and hydroxides forms in the upper part of the weathering crust, while the amount of silica decreases. Low-mobility elements (i.e. Si, Al and Fe) react on the phase separation surface, causing the formation of clay minerals (i.e. vermiculite, montmorillonite) or Al and Fe hydroxides (e.g. goethite).
4. The duration of weathering is associated with an increase in the content of PTEs in solid materials: multifold in the case of Cr (15), Ba (9), Pb (7), Zn (6) and considerably lower in the case of V (1.3), Sr (0.8) and Co (0.4).
5. PTEs co-occur in several chemical fractions in the weathering crust. The highest share was found for cations incorporated in the crystal lattice of minerals and bound by strong crystalline bonds (F4 46%). The lowest share was observed for the reducible fraction (F2 9%). The share of the oxidizable fraction (F3) was 29%, and that of the most mobile and easiest to leach (F1) only 16% of the total concentration.
6. Based on the content of the readily soluble fraction of PTEs, it was concluded that the degree of weathering does not increase the environmental risk, but actually reduces it for Cr, Cr and Pb.
7. The obtained Ecological Risk Index (*ERI*) values indicate that the ecological risk associated with the elements tested is low for the entire area. This means that natural weathering processes do not have any impact on environmental pollution.

## Data Availability

The datasets used and/or analysed during the current study available from the corresponding author on reasonable request.

## References

[1] Price JR, Velbel MA (2003). Chemical weathering indices applied to weathering profiles developed on heterogeneous felsic metamorphic parent rocks. Chem. Geol..

[2] Arif M, Liu G, Yousaf B, Ahmed R, Irshad S, Ashraf A, Zia-ur-Rehman M, Rashid MS (2021). Synthesis, characteristics and mechanistic insight into the clays and clay minerals-biochar surface interactions for contaminants removal—A review. J. Clean. Prod..

[3] Bolewski A, Manecki A (1993). Detailed Mineralogy.

[4] Boixadera J, Poch RM, Garcı´a-Gonza´lez MT, Vizcayno C (2003). Hydromorphic and clay-related processes in soils from the Llanos de Moxos (northern Bolivia). Catena.

[5] Hong H, Ji K, Hei H, Wang C, Liu C, Zhao L, Lanson B, Zhao C, Fang Q, Algeo TJ (2023). Clay mineral evolution and formation of intermediate phases during pedogenesis on picrite basalt bedrock under temperate conditions (Yunnan, southwestern China). Catena.

[6] Caillaud J, Proust D, Philippe S, Fontaine C, Fialin M (2009). Trace metals distribution from a serpentinite weathering at the scales of the weathering profile and its related weathering microsystems and clay minerals. Geoderma.

[7] Gupta N, Kumar YK, Kumar V, Kumar S, Chadd RP, Kumar A (2019). Trace elements in soil-vegetables interface: Translocation, bioaccumulation, toxicity and amelioration—A review. Sci. Total Environ..

[8] Gupta N, Kumar YK, Kumar V, Krishnan S, Kumar S, Nejad ZD, Khan MAM, Alam J (2021). Evaluating heavy metals contamination in soil and vegetables in the region of North India: Levels, transfer and potential human health risk analysis. Environ. Toxicol. Pharmacol..

[9] Gupta N, Kumar YK, Kumar V, Cabral-Pinto MMS, Alam M, Kumar S, Prasad S (2021). Appraisal of contamination of heavy metals and health risk in agricultural soil of Jhansi city, India. Environ. Toxicol. Pharmacol..

[10] Prudencio MI, Sequeira Braga MA, Paquet H, Waerenborgh JC, Pereira LCJ, Gouveia MA (2002). Clay mineral assemblages in weathered basalt profiles from central and southern Portugal: Climatic significance. CATENA.

[11] Zhang Z, Li H, Chi R, Long F, Chi X, Chen W, Chen Z (2022). Inhibition on the swelling of clay minerals in the leaching process of weathered crust elution-deposited rare earth ores. Appl. Clay Sci..

[12] Dixon JL, von Blanckenburg F (2012). Soils as pacemakers and limiters of global silicate weathering. Comptes Rendus Geosci..

[13] Guo J, Pyles C, Krugh W, Negrini R (2019). Clay minerals in the late Quaternary sediment of Tulare Lake, California: Implications for climate change, weathering, and erosion processes. Int. J. Sedim. Res..

[14] Oliva P, Viers J, Dupré B (2003). Chemical weathering in granitic environments. Chem. Geol..

[15] Stoch L (1974). Clay Minerals.

[16] Duzgoren-Aydin NS, Aydin A, Malpas J (2002). Distribution of clay minerals along a weathered pyroclastic profile, Hong Kong. Catena.

[17] Perri F (2020). Chemical weathering of crystalline rocks in contrasting climatic conditions using geochemical proxies: An overview. Palaeogeogr. Palaeoclimatol. Palaeoecol..

[18] Fang Q, Lu A, Hong H, Kuzyakov Y, Algeo TJ, Zhao L, Olshansky Y, Moravec B, Barrientes DM, Chorover J (2023). Mineral weathering is linked to microbial priming in the critical zone. Nat. Commun..

[19] Feizi M, Jalali M, Antoniadis V, Shaheen SM, Ok YS, Rinklebe J (2019). Geo- and nano-materials affect the mono-metal and competitive sorption of Cd, Cu, Ni, and Zn in a sewage sludge-treated alkaline soil. J. Hazard. Mater..

[20] Nesbitt HW, Young GM (1984). Prediction of some weathering trends of plutonic and volcanic rocks based on thermodynamic and kinetic considerations. Geochimica et Cosmochimica Acta.

[21] Song Q, Hong H, Algeo TJ, Fang Q, Zhao C, Liu C, Xu Y (2023). Clay mineralogy mediated by pH and chemical weathering intensity of Permian-Triassic boundary K-bentonites at Dongpan (Guangxi, South China). Chem. Geol..

[22] Vogt T, Clauer N, Larqué P (2010). Impact of climate and related weathering processes on the authigenesis of clay minerals: Examples from circum-Baikal region, Siberia. Catena.

[23] Vorhies J, Gaines R (2009). Microbial dissolution of clay minerals as a source of iron and silica in marine sediments. Nat. Geosci..

[24] White AF, Blum AE, Bullen TD, Vivit DV, Schulz M, Fitzpatric J (1999). The effect of temperature on experimental and natural chemical weathering rates of granitoid rocks. Geochimica Cosmochimica Acta.

[25] Radvanec M, Grecula P, Žák K (2004). Siderite mineralization of the Gemericum superunit (Western Carpathians, Slovakia): Review and a revised genetic model. Ore Geol. Rev..

[26] www.eurotalc.sk/, accessed: 8 March 2023

[27] Davidson CA, Duncan AL, Littlejohn D, Ure AM, Garden LM (1998). A critical evaluation of the tree-stage BCR extraction procedure to assess the potential mobility and toxicity of heavy metals in industrially-contaminated land. Analytica Chimica Acta.

[28] Quevauviller P (2003). Book review. Methodologies for soil and sediment fractionation studies. Sci. Total Environ..

[29] PN-ISO 10390 Polish Norm. Soil quality - pH determination.

[30] Kicińska A, Wikar J (2021). Ecological risk associated with agricultural production in soils contaminated by the activities of the metal ore mining and processing industry—Example from southern Poland. Soil Tillage Res..

[31] Li F, Yu T, Huang Z, Jiang T, Wang L, Hou Q, Tang Q, Liu J, Yang Z (2022). Leaching experiments and risk assessment to explore the migration and risk of potentially toxic elements in soil from black shale. Sci. Total Environ..

[32] Liu Y, Molinari S, Dalconi MC, Valentini L, Ricci G, Carrer C, Ferrari G, Artioli G (2023). The leaching behaviours of lead, zinc, and sulphate in pyrite ash contaminated soil: Mineralogical assessments and environmental implications. J. Environ. Chem. Eng..

[33] Hong H, Fang Q, Cheng L, Wang C, Churchman GJ (2016). Microorganism-induced weathering of clay minerals in a hydromorphic soil. Geochimica et Cosmochimica Acta.

[34] Egli M, Mirabella A, Sartori G (2008). The role of climate and vegetation in weathering and clay mineral formation in late Quaternary soils of the Swiss and Italian Alps. Geomorphology.

[35] Kicińska A (2019). Environmental risk related to presence and mobility of As, Cd and Tl in soils in the vicinity of a metallurgical plant—Long-term observations. Chemosphere.

[36] Li Y, Padoan E, Ajmone-Marsan F (2021). Soil particle size fraction and potentially toxic elements bioaccessibility: A review. Ecotoxicol. Environ. Saf..

[37] Oeser RA, von Blanckenburg F (2020). Do degree and rate of silicate weathering depend on plant productivity?. Biogeosciences.

[38] Hashimoto Y, Kanke Y (2018). Redox changes in speciation and solubility of arsenic in paddy soils as affected by sulphur concentrations. Environ. Pollut..

[39] Kicińska A, Pomykała R, Izquierdo-Diaz M (2022). Changes in soil pH and mobility of heavy metals in contaminated soils. Eur. J. Soil Sci..

[40] Palansooriya KN, Shaheen SM, Chene SS, Tsange DCW, Hashimotof Y, Houg D, Bolanh NS, Rinklebeb J, Ok YS (2020). Soil amendments for immobilization of potentially toxic elements in contaminated soils: A critical review. Environ. Int..

[41] Parviainen A, V’azquez-Arias A, Peinado FJM (2022). Mineralogical association and geochemistry of potentially toxic elements in urban soils under the influence of mining. Catena.

[42] Rodríguez-Hernández A, Lázaro I, Razo I, Briones-Gallardo R (2021). Geochemical and mineralogical characterization of stream sediments impacted by mine wastes containing arsenic, cadmium and lead in North-Central Mexico. J. Geochem. Explor..

[43] Valadares da Silva AP, Silva AO, Dias de Lima FR, Benedet L, Franco A, de Souza JK, Carvalho A, Rodrigues BÉ, Vasconcellos IA, Curi N, Guimarães Guilherme LR, Carbone Carneiro MA (2022). Potentially toxic elements in iron mine tailings: Effects of reducing soil pH on available concentrations of toxic elements. Environ. Res..

[44] Jia Z, Wang J, Zhou X, Zhou Y, Li Y, Li B, Zhou S (2020). Identification of the sources and influencing factors of potentially toxic elements accumulation in the soil from a typical karst region in Guangxi, Southwest China. Environ. Pollut..

[45] Sá F, Menolli LC, Schettini CE, da Silva CA, Caiado CR, de Oliveira Gomes LE, Lima AT, Fraga BA, Rodrigues NR (2021). Time-sequence development of metal(loid)s following the 2015 dam failure in the Doce river estuary, Brazil. Sci. Total Environ..

[46] Wang W, Lin C, Jiang R, Liu Y, Sun X, Lin H, Chen J (2022). Distribution, source identification and environmental risk assessment of potentially toxic elements (PTEs) in the surface sediment of Sanmen Bay, Zhejiang Province, China. Mar. Pollut. Bull..

[47] Deng K, Yang S, Guo Y (2022). A global temperature control of silicate weathering intensity. Nat. Commun..

[48] Chen T, Wen XC, Zhang LJ, Tu SC, Zhang JH, Sun RN, Yan B (2022). The geochemical and mineralogical controls on the release characteristics of potentially toxic elements from lead/zinc (Pb/Zn) mine tailings. Environ. Pollut..

[49] Cabral Pinto MMS, Silva MMVG, Ferreira da Silva EA, Dinis PA, Rocha F (2017). Transfer processes of potentially toxic elements (PTE) from rocks to soils and the origin of PTE in soils: A case study on the island of Santiago (Cape Verde). J. Geochem. Explor..

[50] Cox SF, Rollinson G, McKinley JM (2017). Mineralogical characterisation to improve understanding of oral bioaccessibility of Cr and Ni in basaltic soils in Northern Ireland. J. Geochem. Explor..

[51] Chen JM, Gong G, Zhao LQ, Sun XC (2000). Determination of cation exchange capacity of expansive soils. Rock Mineral Anal..

[52] Feng XH, Zhai LM, Tan WF, Liu F, He JZ (2007). Adsorption and redox reactions of heavy metals on synthesized Mn oxide minerals. Environ. Pollut..

[53] Kabata-Pendias A, Pendias H (1999). Biogeochemistry of Trace Elements.

[54] Fernandes AR, Santos de Souzaa E, de Souza Braza AM, Biranib SM, Ferracciú Alleonic LR (2018). Quality reference values and background concentrations of potentially toxic elements in soils from the Eastern Amazon, Brazil. J. Geochem. Explor..

[55] Gomes PC, Fontes MPF, da Silva AG, Mendonça ES, Netto AR (2001). Selectivity sequence and competitive adsorption of heavy metals by Brazilian soils. Soil Sci. Soc. Am. J..

[56] Hulisz P, Różański SŁ, Boman A, Rauchfleisz M (2022). Can acid sulfate soils from the southern Baltic zone be a source of potentially toxic elements (PTEs)?. Sci. Total Environ..

[57] Kicińska A. 2012. Geochemical diversification of the Beskid Sądecki Mts. and its impact on the transport of selected elements, Disserations Monorgaphs 252, AGH Press [in Polish]

[58] Regulation on the Method of Conducting the Assessment of Surface Pollution. (2016) Dz. U. 2016, it. 1395.

[59] Kicińska A, Smreczak B, Jadczyszyn J (2019). Soil bioavailability of Cadmium, Lead, and Zinc in the areas of Zn-Pb Ore mining and processing (Bukowno, Olkusz). J. Ecol. Eng..

[60] Bajanik S., Ivanicka J., Mello J., Pristas J., Reichwalder P., Snopko L., Vozar J., Vozarova A. 1984. Geological map of Slovakia M 1:50,000 [online]. Bratislava: State Geological Institute of Dionýz Štúr, 2023. http://apl.geology.sk/gm50js.

